# Improving hepatocellular carcinoma diagnosis using an ensemble classification approach based on Harris Hawks Optimization

**DOI:** 10.1016/j.heliyon.2023.e23497

**Published:** 2023-12-09

**Authors:** LiuRen Lin, YunKuan Liu, Min Gao, Amin Rezaeipanah

**Affiliations:** a Department of Pharmacy and Machinery, Qujing Second People's Hospital, Yunnan, Qujing, 655000, China; b Yunnan University of Chinese Medicine, Yunnan Key Laboratory of External Drug Delivery System and Preparation Technology in Universities, Yunnan, Kunming, 650500, China; c Faculty of Life Science and Technology, Kunming University of Science and Technology, Yunnan, Kunming, 650500, China; d Department of Computer Engineering, Persian Gulf University, Bushehr, Iran

**Keywords:** Medical diagnosis system, Ensemble classification, Feature selection, HCC, HHO

## Abstract

Hepato-Cellular Carcinoma (HCC) is the most common type of liver cancer that often occurs in people with chronic liver diseases such as cirrhosis. Although HCC is known as a fatal disease, early detection can lead to successful treatment and improve survival chances. In recent years, the development of computer recognition systems using machine learning approaches has been emphasized by researchers. The effective performance of these approaches for the diagnosis of HCC has been proven in a wide range of applications. With this motivation, this paper proposes a hybrid machine learning approach including effective feature selection and ensemble classification for HCC detection, which is developed based on the Harris Hawks Optimization (HHO) algorithm. The proposed ensemble classifier is based on the bagging technique and is configured based on the decision tree method. Meanwhile, HHO as an emerging meta-heuristic algorithm can select a subset of the most suitable features related to HCC for classification. In addition, the proposed method is equipped with several strategies for handling missing values and data normalization. The simulations are based on the HCC dataset collected by the Coimbra Hospital and University Center (CHUC). The results of the experiments prove the acceptable performance of the proposed method. Specifically, the proposed method with an accuracy of 97.13 % is superior in comparison with the equivalent methods such as LASSO and DTPSO.

## 1 Introduction

Hepato-Cellular Carcinoma (HCC) is one of the dangerous diseases that arises from the tissue of the liver and bile ducts [1,2]. The liver is the largest internal organ and manages hundreds of vital body functions such as helping to eliminate waste, absorb nutrients and manage metabolism. When this cancer occurs, the liver cells are gradually destroyed and their function faces a serious problem [3]. HCC is not very common, but it is one of the cancers that has rising statistics and is steadily increasing [4]. It is estimated that about 1 % of the American population will be affected by this type of cancer during their lifetime [[5], [6], [7]]. In general, factors such as aging, alcohol consumption, smoking, aflatoxin and anabolic steroid use increase the risk of this type of cancer [8].

HCC cannot be detected by routine blood tests and there is only one specific blood test to diagnose it [9,10]. This test is known as Alpha Feto Protein (AFP) and is not available to everyone. On the other hand, only about half of tumors show high levels of AFP. Therefore, a normal AFP test may also not show the presence of HCC. Meanwhile, the issue of HCC diagnosis becomes more complicated considering the fact that AFP is also produced by hepatocyte proliferation [10]. Therefore, AFP levels are likely to be high in individuals with cirrhosis. In general, an increase in AFP level is more indicative of HCC. However, individuals with cirrhosis with no detectable symptoms and normal AFP are still at risk of developing HCC [1,7]. In addition to the AFP test, imaging tests such as abdominal ultrasound, Computed Tomography (CT) scan, and Magnetic Resonance Imaging (MRI) of the liver can be used to diagnose HCC [11].

Another diagnostic test for this type of cancer is Biopsies [12]. Also, if you are at risk of HCC due to cancer risk factors, your doctor may recommend regular screening [13,14]. As with many types of cancer, if HCC is diagnosed in the early stages, there are more treatment options and a better chance of recovery. Risk factors that increase HCC are well known and doctors are able to identify and treat people who are at risk earlier and in the early stages according to scientific criteria. With the aforementioned methods, in addition to cancer diagnosis, its spread rate is also determined [15]. However, all these methods are often not available and do not provide the diagnosis of HCC with high accuracy [16]. Methods that have high diagnostic accuracy are often expensive and only available in some countries.

Currently, machine learning techniques are one of the most important technologies that are used in various fields such as medicine [7]. Today, intelligent medical diagnosis systems using machine learning have attracted the attention of researchers. With using these systems, various diseases such as cancer can be diagnosed more accurately [1]. Often these systems are used as medical aids and help doctors make better diagnoses. These methods diagnose diseases automatically, faster and more accurately, which significantly reduces the duration and costs of treatment [17]. Although intelligent medical diagnosis systems are effective in accurately diagnosing diseases, there are also obstacles and challenges [18]. For example, if sufficient and high-quality data are not available, the accuracy of machine learning techniques will decrease. Interpreting the complexity of machine learning techniques is another challenge. Choosing a suitable algorithm and developing them according to the available data is challenging for researchers.

With this motivation, in this paper, a hybrid machine learning approach including effective feature selection and ensemble classification is proposed for HCC detection. Due to the widespread application of evolutionary algorithms, we apply Harris Hawks Optimization (HHO) as a new evolutionary algorithm to select the subset of effective features. Also, we configure an ensemble classifier to model HCC-related data using a decision tree method. Ensemble classification is developed based on bagging technique. Also, we introduce several strategies for handling missing values and data normalization.

The main contribution of the paper is as follows:

• A hybrid machine learning approach including effective feature selection and ensemble classification is proposed for HCC detection.
• HHO is used as a new evolutionary algorithm to select effective features related to HCC.
• An ensemble classification model is developed using the decision tree method and based on the bagging technique.
• Several strategies for handling missing values and data normalization are introduced.

The rest of the paper is organized as follows: Section 2 deals with related works. Section 3 provides the background of this study. Section 4 explains the details of the proposed method. Section 5 is related to experimental results and comparisons. Finally, the paper ends with the conclusions in Section 6.

## 2 Related works

There are a wide range of studies that propose machine learning-based solutions for HCC detection [1,7,10]. Without loss of generality, researchers show that deep reinforcement learning-based models and ensemble classification models equipped with dimensionality reduction policy are more accurate for HCC detection compared to other models in the literature [10]. In this section, some of the latest available methods with a machine learning perspective are reviewed.

Książek et al. [19] proposed an ensemble classification approach using Evolutionary Computation and Stacking Learning (ECSL) to detect HCC. The authors use seven classic classifiers and four combined classifiers to configure the ensemble classification approach based on stacking learning. Here, a genetic optimization-based approach is implemented for each classifier, where a subset of optimal features is selected for dimensionality reduction. Simulations have been performed on the HCC dataset where the missing values are replaced by a K-Nearest Neighbor (KNN).

Panigrahi et al. [20] introduced two strategies to predict the survival of patients with HCC, one for male patients and the other for female patients. The authors proposed a diverse classifier ensemble of grafted decision tree to model HCC data related to Coimbra's Hospital and University Center (CHUC). Here, a gender survival prediction engine using feature extraction and feature selection techniques is introduced, which the authors claim is the first predictive model for HCC diagnosis. The feature extraction technique includes a Sigmis feature selection scheme that can significantly reduce the dimensions of the data. Also, the feature selection technique is based on a concave minimization feature ranking approach.

Ali et al. [21] proposed the Recursive Feature Elimination with Gradient Boosting (RFE-GB) method to predict liver cancer survival. RFE-GB is applied on the HCC dataset, where missing values are filled based on three strategies including mean, mode, and KNN. The authors use embedded and wrapper techniques for feature selection. These methods are configured using ridge regression in conjunction with logistic regression and Least Absolute Shrinkage and Selection Operator (LASSO). In particular, RFE-GB obtained the best accuracy where the wrapper was used for feature selection. Here, the wrapper can recursively remove redundant features by random forests and gradient boosting.

Książek et al. [22] introduced neighborhood components analysis, genetic algorithm (GA) and support vector machine classifier (NCA-GA-SVM) for modeling HCC data and its prediction. This method is developed based on a new two-level feature selection system. The SVM model embedded in NCA-GA-SVM uses GA to tune default parameters in decision making. Since GA alone does not provide good performance for SVM parameter tuning, the authors used the combination of NCA and GA to solve this problem. Therefore, NCA-GA-SVM configures a two-level algorithm for modeling HCC data, which achieved a classification accuracy of 96.36 %.

Wang et al. [23] presented a model to predict Edmondson-Steiner grade using CEUS data for HCC patients. This model uses ensemble learning and is applied to three CEUS images including arterial, portal vein and delayed phases. Here, segmentation of tumor lesions is done manually. Also, extraction of high-dimensional features is done using Radiomics technique. Meanwhile, the feature selection process to reduce the dimensions of the data is done by T-test and the Least absolute Shrinkage and Selection Operator (LASSO). Finally, the considered features are modeled by an ensemble classifier to predict the Edmondson Steiner classification.

Sharma and Kumar [24] proposed an ensemble learning technique including 15 classical classification models to improve HCC diagnosis. The inputs of this model include clinical trial information, risk factors and geographical information. Each classification model used in the ensemble includes data preprocessing, effective feature selection, and survival classification. Here, each feature is assigned a weight, and features with higher weights are allowed to participate in the ensemble classification model. Algorithms used for feature evaluation include random forest, GA, LASSO Regression (L-1 penalization) and Ridge Regression (L-2 penalization). Meanwhile, the classification models used in ensemble learning include L-1 penalized Gradient Boosting Ensemble Learning (GBEL), L-2 penalized GBEL, RFGBEL, GA optimized GBEL, L-1 penalized RidgeCV (RCV), L-2 penalized RCV, RF-RCV, GA optimized RCV, L-1 penalized Nu-Support Vector Classification (Nu-SVC), L-2 penalized Nu-SVC, RF-NuSVC and GA optimized Nu-SVC. The simulation results show that RFGBEL has a better efficiency compared to other models with an accuracy of 92.93.

Shams et al. [25] presented the Dipper Throated Optimization and Particle Swarm Optimization (DTPSO) method for HCC prediction. DTPSO is configured using four classical classification models such as Linear Discriminant Analysis (LDA), logistic regression, random forest and naive bayes. The output of these models is combined by a Deep Neural Network (DNN) as a meta-model to create the final model. DTPSO uses the characteristics of both Particle Swarm Optimization (PSO) and Dipper Throated Optimization (DTO) methods to provide more accurate prediction of HCC. The effectiveness of DTPSO has been confirmed with an accuracy of 98.52 % on the HCC dataset.

Anisha et al. [26] proposed an Ensemble Learning-based Classification Model (ELCM) for HCC detection equipped with an automatic feature selection approach. This method extracts deep features from CT images using deep Convolutional Neural Network (CNN) models. Here, InceptionResnetV2 and densenet201 models are used as pre-trained deep CNN. The authors use a combination of GA and Ant Colony Optimization (ACO) algorithms to reduce the feature space. Eventually, a heterogeneous ensemble classification model was applied on the extracted features to predict HCC samples in four classes such as metastasis, hepatocellular carcinoma, liver cirrhosis or liver abscess. The simulation results reported an accuracy of 98.3 % for this method.

## 3 Background

In this section, the basic concepts related to the problem of HCC diagnosis and its solution method are presented. The problem of HCC detection is formulated as a classification problem, which requires the use of feature selection methods before modeling due to the high dimensions of the data associated with it. Evolutionary approaches are among the popular strategies for solving the feature selection problem. According to the aforementioned concepts, in this section, some basic explanations related to HCC dataset, classification, feature selection, and evolutionary approaches are presented.

### 3.1 HCC dataset

The HCC dataset was collected by CHUC in 2015 [27]. At CHUC, as one of the largest Portuguese hospitals, 165 patients with HCC were sampled to collect this dataset. The HCC dataset includes 49 features of laboratory, risk factors, demographic and overall survival. All these features were extracted based on the European Association for the Study of the Liver - European Organization for Research and Treatment of Cancer (EASL-EORTC) Clinical Practice Guidelines.

Out of 49 features in HCC dataset, 26 features have qualitative values and 23 features have quantitative values. In total, 10.22 % of the data in this dataset is missing, and only 8 samples have complete values. Samples in this set are labeled based on survival at 1 year, where ‘0’ is coded as “dies” and ‘1’ as “lives”. Overall, 63 samples are labeled ‘0’ and 102 samples are labeled ‘1’. Full details of this dataset, including feature name, type/scale, data range, mean, standard deviation, and missingness are given in Table 1.

**Table 1:** HCC dataset details.

Index	Feature name	Type/scale	Data range	Mean	Standard deviation	Missingness (%)
1	Gender	Qualitative/dichotomous	0/1	0.806	0.397	0
2	Symptoms	Qualitative/dichotomous	0/1	0.639	0.482	10.91
3	Alcohol	Qualitative/dichotomous	0/1	0.739	0.440	0
4	Hepatitis B Surface Antigen	Qualitative/dichotomous	0/1	0.108	0.312	10.3
5	Hepatitis B E Antigen	Qualitative/dichotomous	0/1	0.008	0.089	23.64
6	Hepatitis B Core Antibody	Qualitative/dichotomous	0/1	0.270	0.445	14.55
7	Hepatitis C Virus Antibody	Qualitative/dichotomous	0/1	0.218	0.414	5.45
8	Cirrhosis	Qualitative/dichotomous	0/1	0.903	0.297	0
9	Endemic Countries	Qualitative/dichotomous	0/1	0.079	0.271	23.64
10	Smoking	Qualitative/dichotomous	0/1	0.508	0.502	24.85
11	Diabetes	Qualitative/dichotomous	0/1	0.346	0.477	1.82
12	Obesity	Qualitative/dichotomous	0/1	0.129	0.336	6.06
13	Hemochromatosis	Qualitative/dichotomous	0/1	0.049	0.217	13.94
14	Arterial Hypertension	Qualitative/dichotomous	0/1	0.364	0.483	1.82
15	Chronic Renal Insufficiency	Qualitative/dichotomous	0/1	0.123	0.329	1.21
16	Human Immunodeficiency Virus	Qualitative/dichotomous	0/1	0.020	0.140	8.48
17	Nonalcoholic Steatohepatitis	Qualitative/dichotomous	0/1	0.056	0.231	13.33
18	Esophageal Varices	Qualitative/dichotomous	0/1	0.611	0.490	31.52
19	Splenomegaly	Qualitative/dichotomous	0/1	0.560	0.498	9.09
20	Portal Hypertension	Qualitative/dichotomous	0/1	0.714	0.453	6.67
21	Portal Vein Thrombosis	Qualitative/dichotomous	0/1	0.222	0.417	1.82
22	Liver Metastasis	Qualitative/dichotomous	0/1	0.224	0.418	2.42
23	Radiological Hallmark	Qualitative/dichotomous	0/1	0.681	0.468	1.21
24	Age at diagnosis	Quantitative/ratio	20–93	64.691	13.32	0
25	Grams of Alcohol per day	Quantitative/ratio	0–500	71.009	76.278	29.09
26	Packs of cigarettes per year	Quantitative/ratio	0–510	20.464	51.565	32.12
27	Performance status	Qualitative/ordinal	0–4	1.018	1.182	0
28	Encephalopathy degree	Qualitative/ordinal	1–3	1.159	0.428	0.61
29	Ascites degree	Qualitative/ordinal	1–3	1.442	0.686	1.21
30	International Normalized Ratio (INR)	Quantitative/ratio	0.84–4.82	1.422	0.478	2.42
31	Alpha-FetoProtein (ng/mL)	Quantitative/ratio	1.2–1,810, 346	19299.95	149098.33	4.85
32	Hemoglobin (g/dL)	Quantitative/ratio	5–18.7	12.879	2.145	1.82
33	Mean Corpuscular Volume (fl)	Quantitative/ratio	69.5–119.6	95.120	8.406	1.82
34	Leukocytes (G/L)	Quantitative/ratio	2.2–13000	1473.962	2909.11	1.82
35	Platelets (G/L)	Quantitative/ratio	1.71–459000	113206.44	107118.63	1.82
36	Albumin (mg/dL)	Quantitative/ratio	1.9–4.9	3.446	0.685	3.64
37	Total Bilirubin (mg/dL)	Quantitative/ratio	0.3–40.5	3.088	5.499	3.03
38	Alanine transaminase (U/L)	Quantitative/ratio	11–420	67.093	57.540	2.42
39	Aspartate transaminase (U/L)	Quantitative/ratio	17–553	96.383	87.484	1.82
40	Gamma-glutamyl transferase (U/L)	Quantitative/ratio	23–1575	268.027	258.750	1.82
41	Alkaline phosphatase (U/L)	Quantitative/ratio	1.28–980	212.212	167.944	1.82
42	Total Proteins (g/dL)	Quantitative/ratio	3.9–102	8.961	11.726	6.67
43	Creatinine (mg/dL)	Quantitative/ratio	0.2–7.6	1.127	0.956	4.24
44	Number of Nodules	Quantitative/ratio	0–5	2.736	1.798	1.21
45	Major dimension of nodule (cm)	Quantitative/ratio	1.5–22	6.851	5.095	12.12
46	Direct Bilirubin (mg/dL)	Quantitative/ratio	0.1–29.3	1.930	4.210	26.67
47	Iron (mcg/dL)	Quantitative/ratio	0–224	85.599	55.699	47.88
48	Oxygen Saturation (%)	Quantitative/ratio	0–126	37.029	28.994	48.48
49	Ferritin (ng/mL)	Quantitative/ratio	0–2230	438.998	457.114	48.48

### 3.2 Classification

One of the sciences that is very popular in the present era is data mining [28]. In general, data mining means exploring data that is used in different ways to obtain patterns and gain knowledge. One of the most widely used data mining techniques is classification, which is a supervised learning method [28]. Supervised learning algorithms can apply what has been learned in the past to predict future events. Basically, classification techniques are used to determine the class label of each sample of the dataset. For this purpose, first a classification model is trained on the available data and then the trained model is used for the prediction task [1].

Classification uses mathematical techniques such as decision tree, linear programming, neural network and statistics [29]. For example, decision trees are a tool in decision analysis that uses trees to model data. The decision tree consists of a number of nodes and branches in which it classifies the samples in a way that grows from the root downwards and finally reaches the leaf nodes. Each non-leaf node is characterized by a feature. Also, the leaves of this tree are defined by a class of answers. Meanwhile, the function of the decision tree is that a root node is on top and its leaves are on the bottom. A sample is entered in the root node and a test is performed in this node to determine which of the child nodes this sample passes to Ref. [28].

ID3 is one of the very simple decision tree algorithms proposed by Quinlan [30]. Also, Quinlan developed the C4.5 algorithm as an evolution of ID3. In C4.5, error-based pruning is applied. Both of these algorithms use gain ratio and entropy to determine the rank of features and place them in the upper levels of the tree [28]. Entropy calculates the degree of irregularity of a dataset. The entropy of the dataset S is calculated by Eq. (1) where P(c) is the proportion of data belonging to class c of the dataset. Also, the gain ratio for selecting a feature for the upper levels of the tree is calculated by Eq. (2).

(1) $$I\left(S\right)=\sum_{c}p\left(c\right).\log_{2}\;p\left(c\right)$$

(2) $$Gain\left(A\right)=I\left(S\right)-\;I_{res}\left(A\right)$$

where $I_{res}\left(A\right)$ represents the residual entropy of the data due to the selection of feature A from the dataset and is calculated by Eq. (3).

(3) $$I_{res}\left(A\right)=-\sum_{A}p\left(a\right)\sum_{c}p\left(c|a\right){.\log}_{2}\;p\left(c|a\right)$$

where a represents the subset of samples with feature A selected, p(a) is the probability of dataset labels for feature A, and p(c|a) is the probability of dataset labels for feature A in class c.

**Fig. 1:**
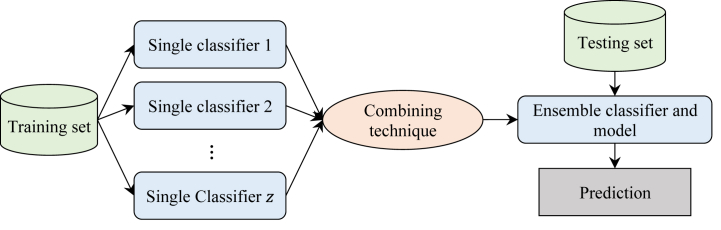
Overview of an ensemble classification model.

Despite the large number of classification models, none of them are the best for all datasets, because their performance varies from one context to another [30]. To improve the performance of classification models, researchers suggested combining the results of several individual classification models. These approaches, known as ensemble classification, use a rule to integrate the results of individual models and create the final classification model. Voting is one of the most common policies for combining the results of individual classification models [28,29]. Fig. 1 shows an overview of an ensemble classification model.

### 3.3 Feature selection

Feature selection is the problem of finding a suitable subset of features among all available features. Typically, the goal of feature selection is to reduce data dimensionality and improve prediction accuracy. In most cases, removing redundant features can lead to a reduction in complexity and an increase in accuracy in classification models. Feature selection solutions seek to find the most appropriate candidate subset among the main features, where the candidate features include $2^{m}$ different candidate subsets. Here, m represents the number of main features from the dataset, and the aim of feature selection is to find $m^{\prime }$ optimal candidate features, where $m^{\prime }<m$.

### 3.4 Evolutionary approaches

**Fig. 2:**
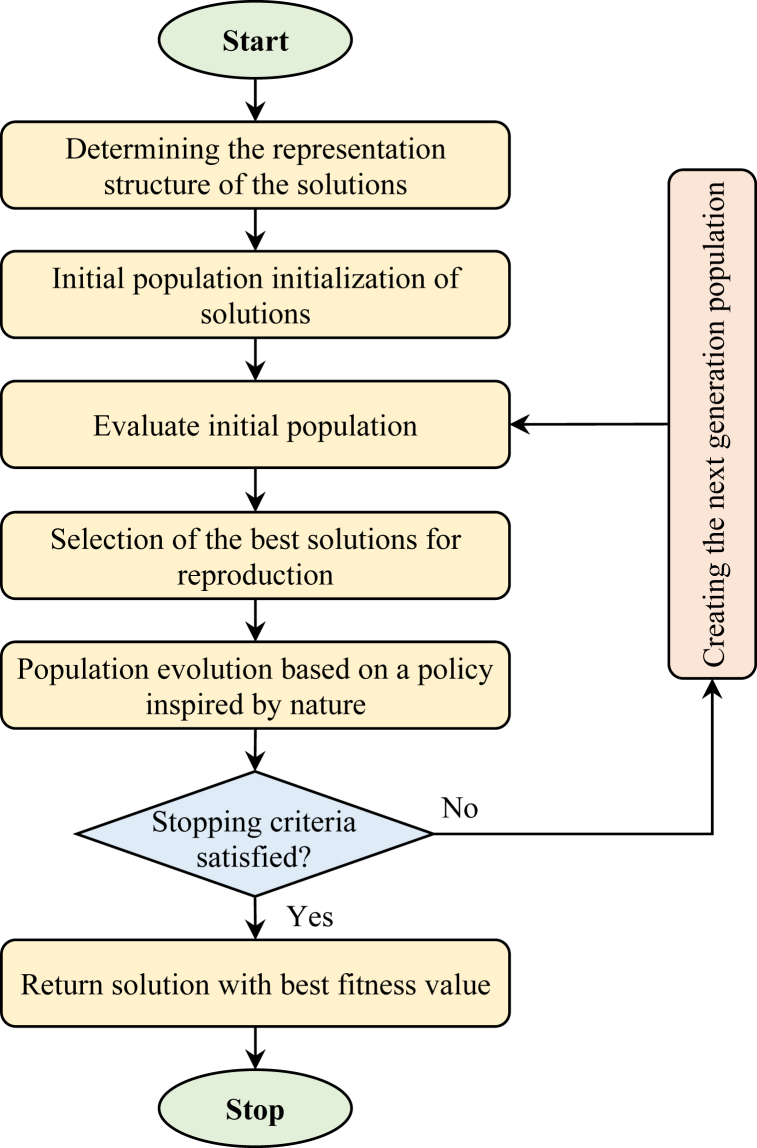
Overview of evolutionary approaches in solving optimization problems.

Evolutionary approach is a common term used for any population-based meta-heuristic optimization algorithm inspired by biological evolution [31]. An overview of the working methodology of evolutionary approaches is shown in Fig. 2. Here, first the initial population is generated and then the solutions are evaluated to select the best ones. After that, the selected solutions are evolved to produce the next generation. Finally, the production of successive generations leads to convergence towards the optimal solution. These approaches are often used to solve problems for which there is no solution in polynomial time. This type of problem is known as Non-deterministic Polynomial time (NP)-hard problems [32]. Different categories of evolutionary approaches have been introduced in recent decades, which can be mentioned as GA, PSO, ACO, Teaching learning-based optimization (TLBO) and HHO [32,33].

Among evolutionary approaches, HHO is presented as a new evolutionary algorithm by Heidari et al. [34], which shows better performance compared to equivalent methods. As a population-based meta-heuristic algorithm, HHO is inspired by cooperative behavior of Harris hawks and also hunting strategy (as surprise pounce) for optimization work. In the hunting strategy, several hawks pounce on a prey from different directions to surprise it. Meanwhile, Harris hawks have several dynamic prey-chasing scenarios that can be modeled mathematically. This modeling is the motivation for designing the HHO algorithm. We have presented the pseudo-code of HHO in Algorithm 1.

**Table:** 

**Algorithm 1.** Pseudo-code of HHO algorithm									
	**Input:** The population size N and maximum number of iterations T.								
	**Output:** The location of rabbit and its fitness value Initialize the random population $X_{i}\left(i=1,2,\ldots ,N\right)$.								
1:	**while**		(stopping condition is not met) **do**						
2:			Calculate the fitness values of hawks.						
3:			Set $X_{rabbit}$ as the location of rabbit (best location).						
4:			**for**		(each hawk ($X_{i}$)) **do**				
5:					Update the initial energy $E_{0}$ and jump strength J: $E_{0}=2rand\left(\right)-1$, J=2(1−rand()).				
6:					Update the E using 2 $E_{0}\left(1-\left(t/T\right)\right)$				
7:					**if**		(|E|≥1) **then//**Exploration phase		
8:							**if**		(q≥0.5) **then**
9:									Update the location vector: $X\left(t+1\right)=X_{rand}\left(t\right)-r_{1}\left|X_{rand}\left(t\right)-2r_{2}X\left(t\right)\right|$
10:							**else**		
11:									Update the location vector: $X\left(t+1\right)=\left(X_{rabbit}\left(t\right)-X_{m}\left(t\right)\right)-r_{3}\left(LB+r_{4}\left(UB-LB\right)\right)$
12:							**end**		
13:					**end**				
14:					**if**		(|E|<1) **then//**Exploitation phase		
15:							**if**		(|E|≥0.5 and r≥0.5) **then//**Soft besiege
16:									Update the location vector: $X\left(t+1\right)=\Delta X\left(t\right)-E\left|JX_{rabbit}\left(t\right)-X\left(t\right)\right|$.
17:							**elseif**		(|E|<0.5 and r≥0.5) **then//**Hard besiege
18:									Update the location vector: $X\left(t+1\right)=X_{rabbit}\left(t\right)-E\left|\Delta X\left(t\right)\right|$.
19:							**elseif**		(|E|≥0.5 and r<0.5) **then//**Soft besiege with progressive rapid dives
20:									Update the location vector: X(t+1)=Y (if F(Y)<F(X(t)) and X(t+1)=Z (if F(Z)<F(X(t)).
21:							**elseif**		(|E|<0.5 and r<0.5) **then//**Hard besiege with progressive rapid dives
22:									Update the location vector: $X\left(t+1\right)=\hat{Y}$ (if $F\left(\hat{Y}\right)<F(X\left(t\right)$) and X(t+1)=Z (if F(Z)<F(X(t)).
23:							**end**		
24:					**end**				
25:			**end**						
53:26:	**end**								
27:	Return $X_{rabbit}$.								

In this algorithm, N is the number of solutions, t is the current iteration and T is the total number of iterations. $X_{i}$ represents the i-th solution from the population and $X_{rabbit}$ refers to the position of rabbit. $E_{0}$ and J show the initial state of its energy and random jump strength of the rabbit in escape procedure, respectively. Also, E stands for dynamic escaping energy. r refers to the chance of a prey in escaping and q indicates the probability of prey identification strategy. X(t) and X(t+1) represent the position vector of hawks in iteration t and t+1, respectively. Here, $r_{1}$ $r_{2}$, $r_{3}$ and $r_{4}$ are random numbers between 0 and 1. LB and UB represent the upper and lower bounds for related variables, respectively. Meanwhile, ΔX(t) represents the difference between the current location and the position vector at iteration t. Here, F is used as the objective function. In addition, Y, Z and $\hat{Y}$ are respectively defined by Eqs. (4), (5), (6).

(4) $$Y=X_{rabbit}\left(t\right)-E\left|JX_{rabbit}\left(t\right)-X\left(t\right)\right|$$

(5) Z=Y+S.LF(D)

(6) $$Y=X_{rabbit}\left(t\right)-E\left|JX_{rabbit}\left(t\right)-X_{m}\left(t\right)\right|$$

where LF represents the levy flight function, D represents the dimensions of the problem, and S represents a random vector. Also, $X_{m}$ is the mean location of all members of the population.

## 4 Proposed method

**Fig. 3:**
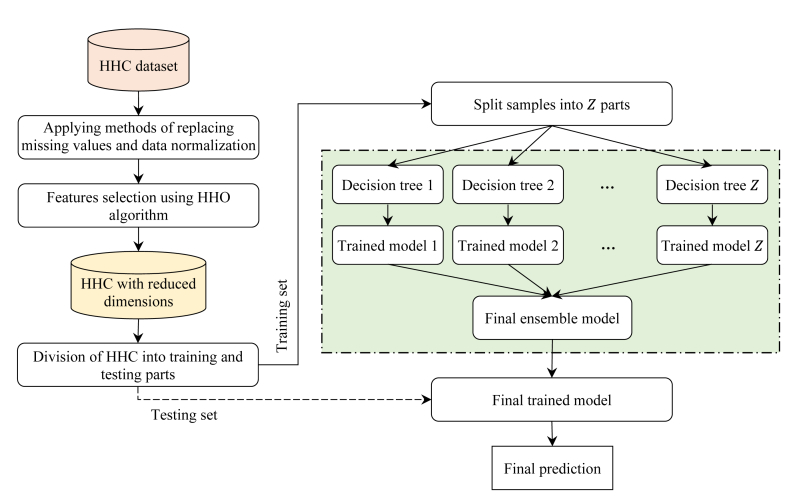
Flowchart of the proposed method.

This paper predicts HCC affected samples using a hybrid machine learning model. The proposed model consists of three main steps, as shown in Fig. 3. The first step is a pre-processing of the associated HCC dataset. In this step, the missing values are replaced by several different methods and then normalization is applied. This step can improve the quality of data for the training process. In the second step, a subset of the most effective features is selected using the HHO algorithm. This step can significantly reduce the complexity of the model by reducing the dimensions of the data. Finally, an ensemble classification model is proposed for HCC diagnosis in the third step. This model is configured using a decision tree and is developed based on the bagging technique.

### 4.1 Data pre-processing

Overall, more than 10 % of the data in the HCC dataset are missing. In fact, only 8 samples of this dataset contain data values for all features. For this reason, it is necessary to replace missing values with appropriate values in the HCC dataset. Here, we introduce several different methods for imputing missing values. These methods include mean, median, highest, zero, one's, and iterative [35]. In mean imputation, average values of each feature are replaced with missing values associated with that feature. In median imputation, the median value obtained for each feature is replaced by the missing values associated with that feature. In highest imputation, missing values are replaced by the highest value in each feature. In zero imputation, any missing value is replaced by the value ‘0’. In one's imputation, any missing value is replaced by the value ‘1’. In iterative imputation, each missing feature value is replaced using a function of other features. Here, the function is configured iteratively with the round robin algorithm [36]. Each of these methods is analyzed and the best method for modeling is considered.

Data normalization is of great importance in data mining, especially when the data are multidimensional with different scales. In this paper, we use the z-score method for the normalization task, where this method maps the scale of features in the range of 0–1 [37]. Eq. (7) shows the process of normalization by z-score method.

(7) $$Z_{i,j}=\frac{x_{i,j}-\mu_{j}}{\sigma_{j}}$$

where $x_{i,j}$ is the true value of sample i of feature j and $Z_{i,j}$ is the z-score normalized value for $x_{i,j}$. Meanwhile, $\mu_{j}$ represents the mean and $\sigma_{j}$ represents the standard deviation for all samples associated with feature j.

### 4.2 Objective function

In this paper, the objective function is formulated by Mean Squared Error (MSE). This objective function is used in both feature selection and classification. Also, we select the best among six missing value replacement methods based on MSE. In mathematics and statistics, MSE is a method of error estimation that shows the difference between estimated and estimated values. Eq. (8) calculates the MSE for a set of N samples.

(8) $$MSE=\frac{1}{N}\sum_{i=1}^{N}{\left(y_{i}-{\hat{y}}_{i}\right)}^{2}$$

where $y_{i}$ represents the actual class label for the i-th sample and ${\hat{y}}_{i}$ represents the predicted class label for the i-th sample.

### 4.3 Feature selection with HHO

HHO Algorithm is a meta-heuristic algorithm recently presented by Heidari et al. [34]. The reason for this naming is that HHO has modeled on Harris hawks to search for and catch prey. In general, the HHO algorithm is modeled in the exploitation and exploration phase and will be described in detail below.

#### 4.3.1 Exploration stage

HHO Algorithm can transfer from exploration to exploitation, and then the exploration behavior changes based on the escape energy of prey. In terms of mathematical escape energy of prey is as Eq. (9).

(9) $$E=2E_{0}\left(1-\frac{t}{T}\right)$$

where $E_{0}$ is the value of the initial energy, which is set by Eq. (10).

(10) $$E_{0}=2r-1\;$$

where t and T represent the current iteration and the highest number of iterations, respectively. $E_{0}$ is the first randomly generated energy and r is a random number in the range [0, 1]. When the escape energy of prey was obtained from the E, the Harris optimizer allowed the Hawk to search in different areas. Conversely, HHO are willing to search locally for the best solutions when the prey energy is E<1. In the exploration phase, the position of the eagle is updated through a random position as Eq. (11).

(11) $$X\left(t+1\right)=\left\{ \begin{array}{c} x_{k}\left(t\right)-r_{1}|x_{k}\left(t\right)-2r_{2}X\left(t\right)|\;q\geq 0.5\\ \left(x_{r}\left(t\right)-x_{m}\left(t\right)\right)-r_{3}\left(LB+r_{4}\left(UB-LB\right)\right)\;q<0.5 \end{array}\right.$$

where X(t+1), X(t), $x_{k}\left(t\right)$, and $(x_{r}\left(t\right)$ represent the next position of Harris Hawk, Hawk's current position, the random position of Harris Hawk, the prey position (the best global solution for the entire population), respectively. In addition, UB and LB are the upper and lower bounds of the search space. Also, $r_{1}$, $r_{2}$, $r_{3}$, $r_{4}$, and q are five independent random numbers of range [0, 1]. Also, $x_{m}$ represents the average position of the current Hawks population and is calculated according to Eq. (12).

(12) $$X_{m}\left(t\right)=\frac{1}{N}\sum_{i=1}^{N}X_{i}\left(t\right)$$

where $X_{m}$ is the m-th largest Hawk in its population and N represents the number of Hawks.

#### 4.3.2 Exploitation stage

Hawk's position in the exploitation phase is updated according to four different types of conditions, which are briefly described below. This behavior is based on the escape energy of the prey (E) and the chance of the prey in successful escape (r<0.5) or in unsuccessful escape (r≥0.5), which is done before the surprise operation.

*Soft Siege:* Soft siege occurs when r≥0.5 and E≥0.5. In this case, Hawk updates its status using Eq. (13).

(13) $$X\left(t+1\right)=\Delta X\left(t\right)-E|{JX}_{r}\left(t\right)-X\left(t\right)|$$

where E represents the escape energy of the prey. Also, X(t+1), t, and ΔX are the hawk's position, the current iteration, and the difference between the position of the prey and the current Hawk's position, respectively. Here, ΔX(t) and J is defined as Eqs. (14), (15), respectively.

(14) $$\Delta X\left(t\right)=X_{r}\left(t\right)-X\left(t\right)$$

(15) J=2(1–rs)

where J is the jump power, and rs is a random number of (0, 1) that changes randomly in each iteration.

*Hard Siege:* Hard siege occurs when r≥0.5 and E<0.5. In this case, the position of Harris hawk is defined as Eq. (16).

(16) $$X\left(t+1\right)=X_{r}\left(t\right)-E\left|\Delta X\left(t\right)\right|$$

where X represents the position of the Hawk, $X_{r}$ shows the position of prey, E is the escape energy of prey, and ΔX indicates the distance difference between the position of prey and Hawk.

*Soft siege along with fast progressive dives:* Soft siege with fast dives occurs when r<0.5 and |E|≥0.5. Hawk gradually chooses the best possible dive to catch the fleeing prey. Hawk's new position in the mentioned conditions will be as Eqs. (17), (18).

(17) $$Y=X_{r}\left(t\right)-E|\;JX_{r}\left(t\right)-X\left(t\right)|$$

(18) Z=Y+S.LF(D)

where Y and Z represent the two newly generated Hawks, E, J, X and t represent the escape energy, jump strength, hawk position, and current iteration, respectively. As well, $X_{r}$ is the prey's position, D shows the total number of dimensions and S is a random vector with dimensions D. LF is the Levy flight function, the hawk's position at this step is updated as Eq. (19).

(19) $$X\left(t+1\right)=\left\{ \begin{array}{c} Y\;if\;F\left(Y\right)<F\left(X\left(t\right)\right)\\ Z\;if\;F\left(Z\right)<F\left(X\left(t\right)\right) \end{array}\right.$$

*Hard siege along with fast progressive divers:* The last condition is a hard siege with fast dives and occurs when r<0.5 and |E|<0.5. In this case, two new solutions are generated as Eqs. (20), (21).

(20) $$Y=X_{r}\left(t\right)-E\;|{JX}_{r}\left(t\right)-\;X_{m}\left(t\right)|$$

(21) Z=Y+S.LF(D)

where E is escape energy, J is jump strength, $X_{m}$ is the position-average of Hawks in the current iteration population, $X_{r}$ is the position of the prey, D is the total number of dimensions, S is a random vector with dimensions D, and LF is the Levy function. The Hawk's position will be updated as Eq. (22).

(22) $$X\left(t+1\right)=\left\{ \begin{array}{c} Y\;if\;F\left(Y\right)<F\left(X\left(t\right)\right)\\ Z\;if\;F\left(Z\right)<F\left(X\left(t\right)\right) \end{array}\right.$$

where each of the solutions Z and Y which perform better in terms of the objective function is selected as the final solution.

#### 4.3.3 Binary solutions

Hyperbolic tangent or V-shaped function is a vital transfer function that can be used to binary the HHO algorithm. This function is defined according to Eq. (23).

(23) $$V\_shaped\left(x\right)=\left|\;\frac{2}{\pi }arc\;\tan\;\left(\frac{\pi }{2}x\right)\right|$$

where x represents the solution of the HHO Algorithm. After applying the V-shaped transfer function, a random threshold is generated and all solutions are converted to zero and one.

### 4.4 Ensemble classifier based on decision tree

**Fig. 4:**
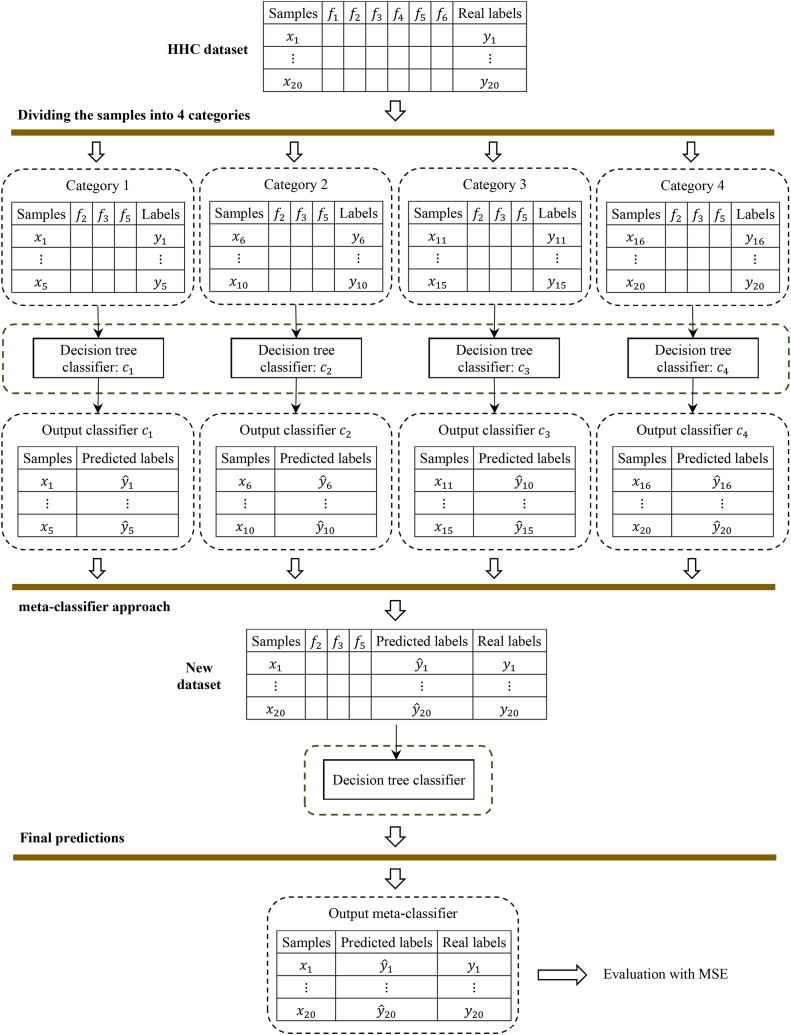
Overview of the ensemble classification algorithm.

After selecting the features and reducing the dimensions of the HCC dataset with the HHO algorithm, we divide the dataset into two parts, training and testing. This is done using the 10-fold cross validation technique [38,39]. After that, the data from the training section is used for modeling and the data from the testing section is used to evaluate the model. In this paper, modeling is done by a homogeneous ensemble classification algorithm. Here, the decision tree model is used for all Z individual classification methods available in ensemble learning. In addition, ensemble classification process is done based on bagging technique. Fig. 4 shows the overview of the proposed ensemble classification algorithm.

The HCC dataset is divided into Z categories, where each category is considered for training by a decision tree model. This example is configured for Z=4 and N=20. Furthermore, we assume that out of the 6 available features, HHO only selects $f_{2}$, $f_{3}$ and $f_{5}$ as effective features. Also, let $c_{1}$, $c_{2}$, $c_{3}$ and $c_{4}$ be the output of decision tree classification models. We collect the output labels together with the selected features into a new dataset. Then the consensus function is applied based on the meta-classifier approach and produces the final classification model. In the meta-classifier approach, the generated dataset is reclassified to create the final model. Finally, this model is used to predict the test set. Actually, meta-classifier uses learning behavior of individual classification models for modeling instead of majority vote. Hence, in case of an error in one of the individual classification models, the meta-classifier model can learn this behavior.

## 5 Experimental results

This section is related to the evaluation of the proposed method for the detection of HCC in comparison with equivalent methods. All simulations were performed on the HCC dataset using MATLAB R2021a software. We implemented the proposed method based on predefined parameters on a machine configured with Intel® Core™ i7-1260P Processor and 32 GB DDR4 Memory. All experiment results for the proposed method are reported based on the average of 20 runs to be reliable.

The rest of this section is as follows. First, imputation methods introduced to handle missing values from the HCC dataset are compared. The best replacement method is configured in the proposed method and used for other experiments. In addition to imputation methods, we examine several classification models to justify the use of decision trees. After that, we analyze the performance of HHO algorithm for feature selection process. We compare several meta-heuristic algorithms to verify the effectiveness of HHO. Finally, the proposed method for modeling the HCC dataset is evaluated. Here, the detection results of HCC patients obtained from the proposed method are compared with several classical classification models as well as several ensemble classification models.

We use Ada-boost, naive bayes, random forest, ID3, logistic regression, support vector machine, and k-nearest neighbor models as classical classifiers [40], as well as use ECSL [19], RFE-GB [21], LASSO [21], DTPSO [25] and ELCM [26] methods as ensemble classifiers for comparison work. We use criteria such as accuracy, precision, recall and F1-score for evaluation and validation [41]. The mathematical definition of these criteria is shown in Eqs. (24), (25), (26), (27) respectively.

(24) $$Accuracy=\frac{TP+TN}{TP+TN+FP+FN}$$

(25) $$\mathrm{Pr}ecision=\frac{TP}{TP+FP}$$

(26) $$Recall=\frac{TP}{TP+FN}$$

(27) $$F1\_score=\frac{2\times Precision\times Recall}{Precision+Recall}$$

where TP is considered as true positive, FP as false positive, TN as true negative, and FN as false negative.

### 5.1 Analysis of missing values imputation

**Fig. 5:**
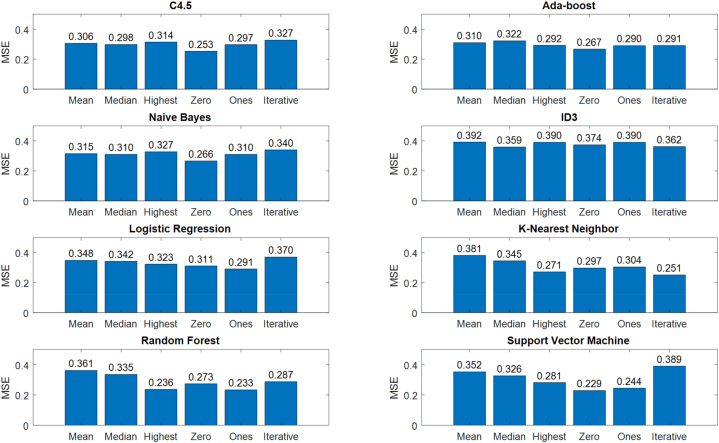
Missing values imputation analysis considering different classification models.

In this paper, several methods were used to impute the missing value on the HCC dataset. These methods include mean, median, highest, zero, one's, and iterative. In this section, we analyzed the performance of the proposed method with each of these methods to find the most suitable method for HCC diagnosis. In addition, we use the C4.5 decision tree as the base classification model in the proposed method. In this section, we show that this model has a better performance for diagnosing HCC patients compared to other models such as Ada-boost, naive bayes, ID3, logistic regression, k-nearest neighbor, random forest and support vector machine. Fig. 5 shows the results of this comparison based on the MSE criterion. The results show that the use of median and zero methods have the best performance for imputing the missing value in HCC. Meanwhile, the proposed ensemble classification model based on Bagging technique is more suitable for HCC data modeling. Also, the results associated with the C4.5 decision tree have better results compared to other classical classification models.

### 5.2 Analysis of HHO algorithm for feature selection

**Fig. 6:**
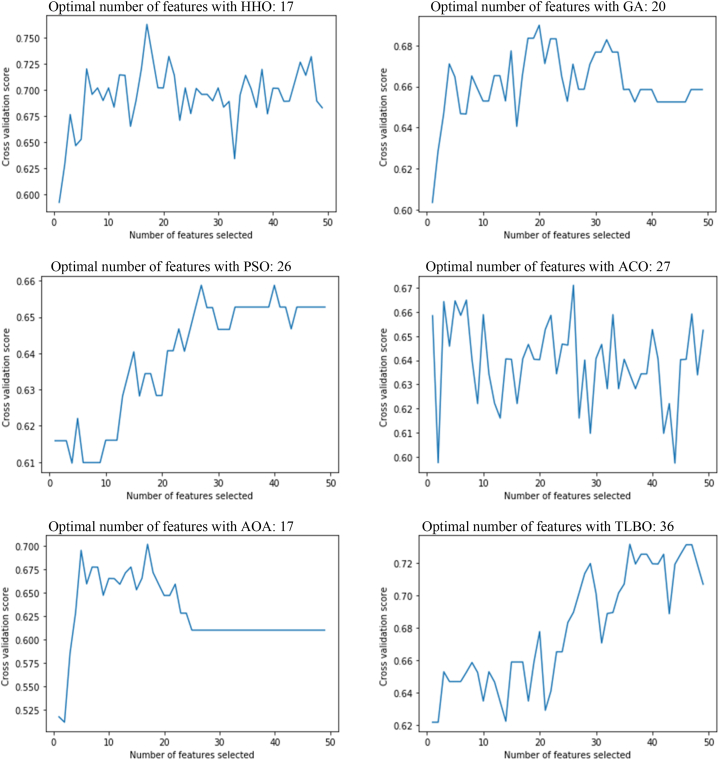
Number of optimal features with different meta-heuristic methods.

Since using the median method to replace missing data as well as the C4.5 decision tree as the base classification model provides the best performance in HCC detection, we apply the proposed method with this configuration in other experiments. In this section, the efficiency of the HHO algorithm for the feature selection process is evaluated. We evaluate the performance of HHO compared to GA, PSO, ACO, AOA and TLBO. First, the cross-validation score for each algorithm is compared when considering the number of different features. The results of this comparison are presented in Fig. 6. Here, the objective is to find the optimal number of features for the classification task.

According to the obtained results, the proposed method using HHO with 16 features has the best performance. The best performance was obtained when using GA for feature selection with 20 features. Also, PSO and ACO provide the best performance considering a subset of 26 and 27 features, respectively. Meanwhile, AOA and TLBO have competitive results with HHO, reporting the optimal number of features with 17 and 36, respectively. According to the obtained results, it can be argued that the number of suitable features is between 17 and 36, and on average 25 or 26 features have a direct effect on the classification output.

**Fig. 7:**
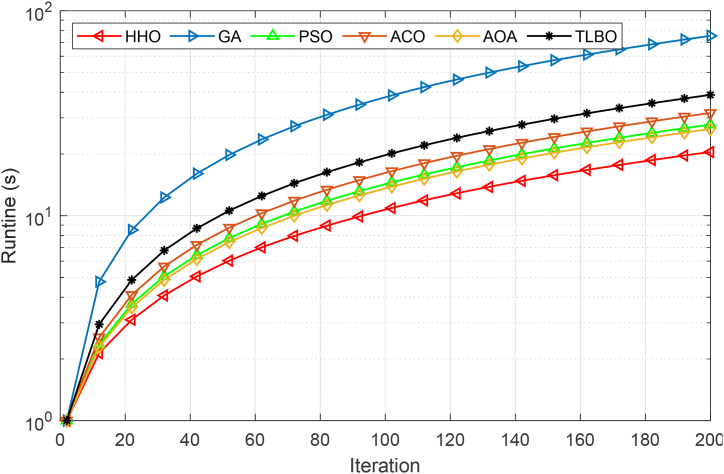
Comparison results of different meta-heuristic methods based on run-time.

The convergence results proved the superiority of HHO in terms of the number of features and cross-validation score, but it is also necessary to compare the run-time of the algorithms. Because the modeling process is related to decision-making, and therefore the complexity of the feature selection algorithm can lead to an increase in the prediction time. With this motivation, we have shown the run-time of different meta-heuristic methods for feature selection in Fig. 7. As illustrated, the run-time of all methods except TLBO is almost similar and there is little difference between them. However, HHO has slightly better run-time compared to other methods. Meanwhile, TLBO has more run-time due to the configuration of the optimization process in two separate phases. This comparison is based on 200 replications considering MSE.

Finally, we have reported the full results of the comparison of HHO with other meta-heuristic methods in Table 2. These results are presented based on different criteria such as accuracy, precision, recall and F1-score. We also provided the standard deviation for each experiment. In general, the results of the comparisons indicate the selection of more suitable features for modeling HCC data by the HHO algorithm. Here, HHO manages to provide excellence on all four different criteria. In addition, we have presented the number of selected features for each algorithm, where HHO has achieved the best results with the least number of features.

**Table 2:** Results related to different meta-heuristic methods in the process of selecting effective features.

Feature selection method	Accuracy	Precision	Recall	F1-score	Number of selected features
GA	0.854 (0.002)	0.843 (0.009)	0.854 (0.011)	0.848 (0.006)	20
PSO	0.894 (0.021)	0.821 (0.021)	0.878 (0.014)	0.849 (0.008)	26
ACO	0.905 (0.016)	0.917 (0.019)	0.862 (0.025)	0.889 (0.005)	27
AOA	0.921 (0.021)	0.945 (0.010)	0.911 (0.021)	0.928 (0.014)	17
TLBO	0.957 (0.009)	0.970 (0.011)	0.971 (0.006)	0.952 (0.019)	36
HHO	0.971 (0.002)	0.965 (0.014)	0.986 (0.021)	0.975 (0.025)	17

### 5.3 Analysis of the ensemble classification model

**Fig. 8:**
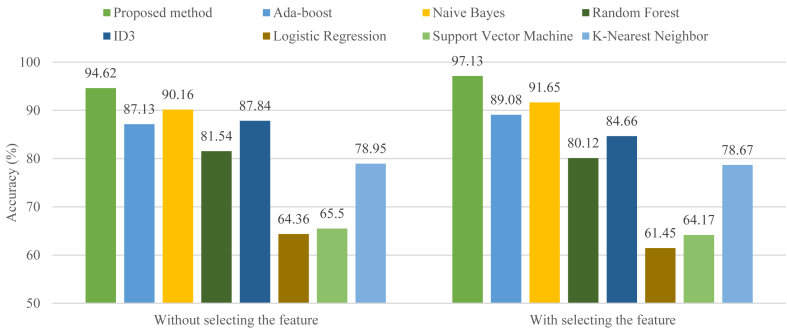
Comparing the results of the proposed ensemble classification method with some classical classification models.

Fig. 8 shows the results obtained from the implementation of the proposed ensemble classification method in comparison with some classical classification models. These models include Ada-boost, naive bayes, random forest, ID3, logistic regression, support vector machine, and k-nearest neighbor [42]. This comparison is reported based on the accuracy criterion [43,44]. In this comparison, the results of the proposed method as well as classical classification models with and without consideration of feature selection are reported. Here, classical classification models are configured based on selected features by the proposed method. The results show that the proposed method has achieved the best classification accuracy in both cases with and without feature selection. Considering the feature selection process, the accuracy of 97.13 % has been achieved by the proposed ensemble classification method compared to other methods. After the proposed method, naive bayes is the second-best classification model with an accuracy of 91.65 %. Meanwhile, the weakest performance belongs to logistic regression algorithm and support vector machine with 61.45 % and 64.17 %, respectively.

In the last experiment, we evaluate the proposed ensemble classification method in comparison with some equivalent methods. These methods include ECSL [19], RFE-GB [21], LASSO [21], DTPSO [25] and ELCM [26]. Table 3 shows the results of this comparison on the HCC dataset based on accuracy, precision, recall and F1-score criteria. Here, the proposed method after ELCM is the best method for predicting HCC patients with accuracy and F1-score equal to 0.971 and 0.975, respectively. Here, ELCM with an accuracy of 0.982 provided the best results for modeling the HCC data. ELCM uses a hybrid algorithm including GA and ACO based on CNN as well as a heterogeneous ensemble classification technique to model the data. Considering the accuracy results, the proposed method is 8.8 %, 4.2 %, 2.6 %, and 1.3 % superior compared to ECSL, RFE-GB, LASSO, and DTPSO, respectively. Also, compared to ELCM, the proposed method reports about 1.1 % weaker performance.

**Table 3:** Comparison of the proposed ensemble classification method with other ensemble classification methods in the literature.

Ensemble method	Accuracy	Precision	Recall	F1-score
ECSL [19]	0.892 (0.008)	0.881 (0.011)	0.902 (0.005)	0.891 (0.010)
RFE-GB [21]	0.932 (0.001)	0.859 (0.013)	0.944 (0.005)	0.899 (0.019)
LASSO [21]	0.946 (0.006)	0.955 (0.013)	0.936 (0.012)	0.945 (0.016)
DTPSO [25]	0.959 (0.016)	0.943 (0.015)	0.949 (0.016)	0.946 (0.004)
ELCM [26]	0.982 (0.022)	0.978 (0.001)	0.979 (0.023)	0.978 (0.003)
Proposed method	0.971 (0.002)	0.965 (0.014)	0.986 (0.021)	0.975 (0.025)

## 6 Conclusion

Hepatocellular carcinoma is the fifth most common cancer in the world and the third most common cause of death from cancer (after lung and stomach cancer). This cancer may have few symptoms and thus may be detected in advanced stages. Hence, the development of mechanisms for early detection of hepatocellular carcinoma is important to improve the health of society. This paper proposed a hybrid algorithm based on machine learning approaches to address this problem. We used the Harris hawk's optimization algorithm to select the subset of effective features. Also, we developed an ensemble classification model that uses the decision tree method and is configured based on the bagging technique. The proposed method is modeled on the HCC dataset with 165 samples and 49 features. Since this dataset has many missing values, we presented several techniques to handle this problem. These techniques together with data normalization led to the improvement of the initial dataset and thus to the improvement of the training process. The simulation results show that the selection of the median method is more efficient than other methods for imputing missing values in the HCC dataset. Also, the simulation results show that the proposed method with 17 features has reached the best accuracy of 97.13 %, which is better than other existing methods. Since the Harris hawk's optimizer has computational complexity, the low dimensions of the HCC dataset can be expressed as a limitation of the proposed method. Hence, the modeling of other datasets related to hepatocellular carcinoma using the proposed method is worth future studies.

## Data availability statement

All data available upon request.

## Declaration of competing interest

The authors declare that they have no known competing financial interests or personal relationships that could have appeared to influence the work reported in this paper.
